# Supplementary material for: Inappropriate Use of Oral Antithrombotic Combinations in an Outpatient Setting and Associated Risks: A French Nationwide Cohort Study
Source: J Clin Med. 2021 May 27;10(11):2367. doi: 10.3390/jcm10112367 (PMC8198137; doi:10.3390/jcm10112367)
Supplement: Supplementary file 1 [file jcm-10-02367-s001.zip › jcm-1183480-supplementary.pdf]

SUPPLEMENTARY INFORMATION TITLES

Supplemental Table S1: Definitions used to identify comorbid conditions in the *Échantillon généraliste des bénéficiaires* (EGB).

Supplemental Text S1: Definitions used to identify indication for oral antithrombotics combination in the *Échantillon généraliste des bénéficiaires* (EGB).

Supplemental Table S2: Proportion of oral antithrombotic (AT) combinations according to AT treatment group at study entry (appropriate or inappropriate oral AT combinations). Values are number (percentages) unless stated otherwise.

Supplemental Table S3: Prescribers of antithrombotics (ATs). Values are number (percentages) unless stated otherwise.

Supplemental Table S4: All antithrombotics (ATs) deliveries during the study period. Values are number (percentages) unless stated otherwise.

Supplemental Table S5: Cumulative incidence (%, 95% confidence interval) at 5 years of oral antithrombotic (AT) combinations, considering the competitive risk of death, for the whole cohort (N = 22220), using 3 different definitions for AT combinations (sensitivity analysis)

Supplemental Table S6: Duration of oral antithrombotic (AT) combinations (median, 25-75 interquartile range, days) in the whole cohort and stratified by age

Supplemental Table S7: Proportion of oral antithrombotic (AT) combinations with non-recommended indication for use, using 3 different definitions for oral AT combinations (sensitivity analysis). Values are number (percentages) unless stated otherwise.

Supplemental Table S8: Risk of major bleeding in individuals with inappropriate oral AT combinations (N = 2580) versus appropriate oral antithrombotic (AT) combinations (N = 1932) as a reference during their follow up (estimated by the fitted Fine and Gray model with death as competiting event).

Supplemental Table S1: Definitions used to identify comorbid conditions in the *Échantillon généraliste des bénéficiaires* (EGB).

	Hospital discharge diagnoses (ICD-10 codes)	LTD (ICD-10 codes)	Specific procedures (CCAM codes)	Drug reimbursement (ATC codes)
**Inclusion criteria**				
**Antiplatelet**				
Aspirin				B01AC06
Clopidogrel				B01AC04
Prasugrel				B01AC22
Ticagrelor				B01AC24
Dipyridamole				B01AC07
Aspirin and clopidogrel				B01AC30
**Vitamin K antagonist**				
Warfarin				B01AA03
Acenocoumarol				B01AA07
Fluindione				B01AA12
**Direct oral anticoagulant**				
Rivaroxaban				B01AF01
Apixaban				B01AF02
Dabigatran				B01AE07
**Injectable anticoagulant**				
Calciparine				B01AB01
Dalteparine				B01AB04
Enoxaparine				B01AB05
Nadroparine				B01AB06
Tinzaparine				B01AB10
Rivaroxaban 10 mg, dabigatran 75 mg, calciparin 5000 UI, dalteparin 2500 UI and 5000 UI, enoxaparin 2000 UI and 4000 UI, nadroparin 2850 UI, tinzaparin 2500 UI and 4500 UI, fondaparinux 0.3 ml and 0.35 ml were not considered in this study. These treatments, with theses doses, are usually prescribed to prevent thromboembolic diseases and do not represent an increased risk of bleeding				
**Exclusion or censorship criteria**				
Auto-immune disease	K50, K51, M05-M09, M45, M46, L93, L94, M30-M36	K50, K51, M05-M09, M45, M46, L93, L94, M30-M36		
HIV - AIDS	B20-B24, Z21	B20-B24, Z21		

Hemophilia	D66, D67, D68, D69	D66, D67	
Active cancer	D00–D09, C00–C26, C30–C41, C43–C58, C60–C97, D37–D48	D00–D09, C00–C26, C30–C41, C43–C58, C60–C97, D37–D48	
**Major neuro-cardiovascular comorbidities**			
Non-valvular atrial fibrillation	I48	I48	Radiofrequency ablation, cardioversion DERP003; DASF074; DERD001; DENF017; DENF018; DENF021
Valvular heart disease	I34, I35, I36, I05, I06, I08, I09		Valvular heart surgery: DBAF003; DBAF002; DBAF005; DBAF004; DBAF001; DBPA002; DBPA004; DBPA005; DBPA006; DBPA007; DBMA008; DBMA012; DBMA003; DBMA002; DBMA011; DBKA004; DBKA008; DBKA007; DBKA012; DBKA010; DBKA005; DBKA002; DBKA006; DBKA003; DBKA001; DBKA011; DBKA009; DBMA007; DBMA013; DBMA005; DBMA009; DBMA010; DBMA006; DBMA001; DBMA015; DBMA004; DBLF009; DBLF001; DBLA004; DBSF001; DBEA001
Coronary heart disease	I20-I25	I20-I25	Coronary stent: DDAA002; YYYY082; DDAF003; DDAF004; DDAF006; DDAF007; DDAF008; DDAF009; DDPF002<br><br>Coronary artery by-pass graft: DDMA003;DDMA004;DDMA005;DDMA006;DDMA007;DDMA008;DDMA009;DDMA011;DDMA012;DDMA013;DDMA015;DDMA016;DDMA017;DDMA018;DDMA019;DDMA020;DDMA021;DDMA022;DDMA023;DDMA024;DDMA025;DDMA026;DDMA027;DDMA028;DDMA029;DDMA030;DDMA031;DDMA032;DDMA033;DDMA034;DDMA035;DDMA036;DD

			MA037;DDMA038;DDQH006;DDQH014;DDQ H011;DDQH013;DDQH015	
Venous thrombo-embolism	I26; I80 (except I80.0); I81; I82			
History of stroke or arterial systemic embolism	I63 (except I63.6); G46 related to I63 or I69.3; I74; G45	I63; I74; G45		
Peripheral vascular disease	I65, I66, I672, I70 - I73	I70 - I73	Lower limb: EEAF002; EEAF004; EEAF006; EEPF001; EELF002; EECA003<br>Carotid or vertebral arteries: EBAF001; EBAF006 EBAF010; EBAF011; EBAF014; EAAF902	
**Baseline comorbidities**				
Hypertension				Diuretics, beta blockers, calcium channel blockers, agents acting on the renin-angiotensin system and other antiadrenergic agents (at least 3 reimbursements over a year)
Diabetes	E10–E14<br>G590, G632, G730, G990, H280, H360, I792, L97, M142, M146, N083, G590, G632, G730, G990, H280, H360, I792, L97, M142, M146, N083	E10–E14		Insulins and blood glucose lowering drugs (at least 3 reimbursements over a year)
Dyslipidemia				HMG CoA reductase inhibitors, fibrates, ezetimibe (at least 3 reimbursements over a year)

Obesity	E66	E66	
Heart failure	I11.0; I13.0; I13.2; I13.9; I50; K76.1; J81	I50	
Chronic kidney disease	N18;I12.0; I13.1; I13.2; E10.2; E11.2; E13.2; E14.2? N08.3; Z49.0-Z49.2; Z94.0; Z99.2	N18;I12	
Chronic hepatic disease	R18; I85; K70; K71.4; K71.5; K71.7; K72; K73; K74; K76.1; B18; C22; C78.7	K70; K73; K74; B18; C22	
Dementia	F00; F01; F02; F03; F05.1; G30; G31.1	F00; F01; F02; F03; G30	Anti-dementia drugs (at least 3 reimbursements over a year)
History of major bleeding	I60-I62; S063; S064; S065; S066; K250; K252; K254; K256; K260; K262; K264; K266; K270; K272; K274; K276; K280; K282; K284; K286; K290; K920; K921; K922; I850; N02; R31; J942; R040; R041; R042; R048; R049; D62; K661; K624; M250; R58; N920; N921; N924; N938; N939; N950; H113; H356; H431; H450; H922; I312		
Anemia	D50; D51; D52; D53; D55; D56; D57; D58; D59; D60; D62; D63; D64		
COPD	J43; J44;	J43; J44;	
Smoking abuse	F17; Z71.6; Z72.0; J43; J44;	J961; J43; J44;	

Alcohol abuse	F10; K70; T51; K860;<br>G31.2; G62.1; G72.1; I42.6;<br>K29.2; Z71.4; Z72.1; Z50.2

Abbreviations: ATC: Anatomical Therapeutic Chemical classification system; CCAM: Common Classification of Medical Procedures; COPD: chronic obstructive pulmonary disease; EGB: Échantillon Généraliste des bénéficiaires; HIV: human immunodeficiency virus; ICD-10: international classification of diseases, 10th revision; LTD: long-term disease

Supplemental Text S1: Definitions used to identify indication for oral antithrombotics combination in the *Échantillon généraliste des bénéficiaires* (EGB).

Triple therapy (1 oral anticoagulant (vitamin K antagonist or direct oral anticoagulant) + aspirin + clopidogrel)

We searched for indication(s) of oral triple therapy 3 months before the initiation: diagnosis of coronary heart disease with percutaneous coronary intervention (PCI, stent) or coronary artery bypass graft (CABG) AND an indication for oral anticoagulation*

Dual therapy (1 oral anticoagulant (vitamin K antagonist or direct oral anticoagulant) + aspirin or clopidogrel)

We searched for indication(s) of dual therapy 6 months before the initiation: diagnosis of acute coronary syndrome medically managed or with PCI (stent) or with CABG; or PCI (stent); or lower extremity artery disease with revascularization; AND an indication for oral anticoagulation*

Dual antiplatelet therapy (aspirin + clopidogrel)

We searched for indication(s) of dual antiplatelet therapy (aspirin and clopidogrel) 3 months before the initiation: diagnosis of acute coronary syndrome medically managed or with PCI (stent) or with CABG; or PCI (stent) or CABG (without acute coronary syndrome); or lower extremity artery disease with revascularization (stent); or carotid/vertebral extremity artery disease with revascularization; AND no indication for anticoagulation.*

Dual antiplatelet therapy (aspirin + prasugrel)

We searched for indication(s) of dual antiplatelet therapy (aspirin and prasugrel) 3 months before the initiation: diagnosis of acute coronary syndrome with PCI or with CABG AND no indication for anticoagulation* AND no stroke history.

Dual antiplatelet therapy (aspirin + ticagrelor)

We searched for indication(s) of dual antiplatelet therapy (aspirin and ticagrelor) 3 months before the initiation: diagnosis of acute coronary syndrome medically managed or with PCI or with CABG 3 months before the initiation of oral AT combinations; AND no indication for anticoagulation* AND no cerebral bleeding history.

*** Indication for anticoagulation:**

- Mechanical heart valve prosthesis (only vitamin K antagonist, contraindicated for direct oral anticoagulant) within 5 years before initiation
- Non-valvular atrial fibrillation with CHA2DS2-VASC score ≥ 2 for men and ≥ 3 for women within 5 years before the initiation
- Or venous thromboembolic disease with an indication for anticoagulation (≤ 6 months)

To note, individuals > 75 years old always have a CHA2DS2-VASC score ≥ 2 for men and ≥ 3 for women. Individuals with vascular disease have at least a CHA2DS2-VASC score ≥ 1 for men and ≥ 2 for women, and anticoagulation therapy to prevent thromboembolism should be considered. For these reasons, we considered that all individuals in our cohort required anticoagulation if they had non-valvular atrial fibrillation.

All codes used can be found in supplementary material Table 1.

Supplemental Table S2: Proportion of oral antithrombotic (AT) combinations according to AT treatment group at study entry (appropriate or inappropriate oral AT combinations). Values are number (percentages) unless stated otherwise.

	**Appropriate[a] oral AT combination N = 576**	**Inappropriate[a] oral AT combination N = 411**
Dual antiplatelet therapy[b]	563 (98)	340 (83)
Dual therapy[c]	10 (2)	66 (16)
Triple therapy[d]	3 (0.5)	3 (0.7)
Contraindicated oral AT combinations[e]	0 (0)	2 (0.5)

[a] Appropriate or inappropriate oral AT combinations: according to guidelines
[b] Dual antiplatelet therapy: aspirin–clopidogrel, aspirin–ticagrelor, aspirin–prasugrel
[c] Dual therapy: aspirin– VKA, aspirin–DOA, clopidogrel–VKA, clopidogrel–DOA
[d] Triple therapy: aspirin–clopidogrel–VKA, aspirin–clopidogrel–DOA
[e] Contraindicated oral AT combinations: P2Y12 inhibitors combinations, anticoagulant combinations longer than 15 or 30 days, dual therapy or triple therapy with ticagrelor or prasugrel, combinations of 3 antiplatelets
Abbreviations: AT: antithrombotics; VKA: vitamin K antagonist; DOA: direct oral anticoagulant

Supplemental Table S3: Prescribers of antithrombotics (ATs). Values are number (percentages) unless stated otherwise.

	**First oral AT delivery at study entry (N = 22220)**	**First oral AT monotherapy delivery at study entry (N = 21233)**	**First oral AT combination delivery at study entry (N = 987)**	**All oral AT deliveries during the study (N = 426736)**
**Ambulatory setting, n (%)**				
General practitioner	11695 (53)	11523 (54)	172 (17)	353 232 (83)
Cardiologist	3013 (14)	2820 (13)	193 (20)	24 633 (6)
Other specialists	1062 (52)	1050 (5)	32 (3)	9289 (2)
**Hospital physician, n (%)**	6430 (29)	5840 (28)	590 (60)	39 582 (9)

Abbreviations: AT: antithrombotics

Supplemental Table S4: All antithrombotics (ATs) deliveries during the study period. Values are number (percentages) unless stated otherwise.

	**Number of individuals with at least one reimbursement of the AT during the study period (N = 22220)**
**Antiplatelet, n (%)**	
Aspirin	17735 (80)
Clopidogrel	2841 (13)
Ticagrelor	932 (4)
Aspirin and clopidogrel	1113 (5)
Prasugrel	303 (1)
Dipyridamole	14 (0.06)
**Vitamin K antagonist, n (%)**	
Fluindione	1462 (7)
Warfarine	480 (2)
Acenocoumarol	83 (0.4)
**Direct oral anticoagulant, n (%)**	
Rivaroxaban	2207 (10)
Apixaban	1665 (7)
Dabigatran	630 (3)
**Injectable anticoagulant, n (%)**	
Tinzaparin	489 (2)
Enoxaparin	444 (2)
Dalteparin	191 (0.9)
Calciparin	143 (0.6)
Nadroparin	41 (0.2)

Supplemental Table S5: Cumulative incidence (%, 95% confidence interval) at 5 years of oral antithrombotic (AT) combinations, considering the competitive risk of death, for the whole cohort (N = 22220), using 3 different definitions for AT combinations (sensitivity analysis)

	**Principal analysis AT combination ≥ 15 days[a]**	**Sensitivity analysis AT combination ≥ 30 days[b]**	**Sensitivity analysis AT combination ≥ 45 days[c]**
**Oral AT combinations**	N = 4466; **27.8 (26.8 to 28.9)**	N = 3715; **22.4 (21.6 to 23.3)**	N = 3203; **18.6 (17.8 to 19.3)**
**Dual antiplatelet therapy[d]**	N = 3134; 18.7 (17.9 to 19.5)	N = 2844; 16.4 (15.7 to 17.1)	N = 2569; 14.3 (13.7 to 14.9)
**Dual therapy[e]**	N = 1075; 9.1 (8.3 to 9.9)	N = 731; 6.0 (5.4 to 6.6)	N = 543; 4.3 (3.9 to 4.8)
**Triple therapy[f]**	N = 67; 0.8 (0.5 to 1.3)	N = 46; 0.5 (0.2 to 0.8)	N = 34; 0.2 (0.1 to 0.3)

[a] AT combination: ≥ 15 days, competitive risk of death: N = 1090
[b] AT combination: ≥ 30 days, competitive risk of death: N = 1137
[c] AT combination: ≥ 45 days, competitive risk of death: N = 1181
[d] Dual antiplatelet therapy: aspirin–clopidogrel, aspirin–ticagrelor, aspirin–prasugrel
[e] Dual therapy: aspirin–VKA, aspirin– DOA, clopidogrel–VKA, clopidogrel–DOA
[f] Triple therapy: aspirin–clopidogrel–VKA, aspirin–clopidogrel–DOA
Abbreviations: AT: antithrombotics; DOA: direct oral anticoagulant; VKA: vitamin K antagonist

Exposure to oral AT combination was defined as exposure to at least 2 oral ATs for at least 15 successive days. As sensitivity analyses, we defined oral AT combination as exposure to at least 2 different oral ATs for at least 30 or 45 successive days.
For cumulative incidence, only the first oral AT combination of interest per person was used.

Supplemental Table S6: Duration of oral antithrombotic (AT) combinations (median, 25-75 interquartile range, days) in the whole cohort and stratified by age

	All cohort<br>N = 22220	< 65 years<br>N = 9310	≥ 65 years<br>N = 12 910	≥ 80 years<br>N = 4286
**All oral AT combinations**	115 [30–360]	172 [30–390]	90 [30–324]	60 [30–293]
**Dual antiplatelet therapy[a]**	239 [53–412]	270 [60–420]	205 [48–390]	207 [53–399]
**Dual therapy[b]**	42 [27–135]	50 [28–164]	40 [26–123]	30 [25–84]
**Triple therapy[c]**	50 [25–107]	46 [26–159]	51 [25–99]	47 [22–114]

Definition used for oral AT combination: ≥ 15 days, competitive risk of death: N = 1090

[a] Dual antiplatelet therapy: aspirin–clopidogrel, aspirin–ticagrelor, aspirin–prasugrel

[b] Dual therapy: aspirin–VKA, aspirin–DOA, clopidogrel–VKA, clopidogrel–DOA

[c] Triple therapy: aspirin–clopidogrel–VKA, aspirin–clopidogrel–DOA

Abbreviations: AT: antithrombotics; DOA: direct oral anticoagulant; VKA: vitamin K antagonist

Supplemental Table S7: Proportion of oral antithrombotic (AT) combinations with non-recommended indication for use, using 3 different definitions for AT combinations (sensitivity analysis). Values are number (percentages) unless stated otherwise.

	**AT combination ≥ 15 days N = 5945**	**AT combination ≥ 30 days N = 4725**	**AT combination ≥ 45days N = 3848**
**Contraindicated oral AT combinations[a]**	N = 370 (6%)	N = 164 (3%)	N = 84 (2%)
**Dual antiplatelet therapy[b]**	N = 3962<br>Non-recommended indication found:<br>N = 2013 (51%)	N = 3484<br>Non-recommended indication found:<br>N = 1652 (47%)	N = 2985<br>Non-recommended indication found:<br>N = 1234 (41%)
**Dual therapy[c]**	N = 1401<br>Non-recommended indication found:<br>N = 1286 (92%)	N = 934<br>Non-recommended indication found:<br>N = 840 (90%)	N = 667<br>Non-recommended indication found:<br>N = 586 (88%)
**Triple therapy[d]**	N = 212<br>Non-recommended indication found:<br>N = 147 (69%)	N = 143<br>Non-recommended indication found:<br>N = 92 (64%)	N = 112<br>Non-recommended indication found:<br>N = 65 (58%)
**All oral AT combinations**	N = 5945<br>Non-recommended indication found:<br>**N = 3816 (64%)**	N = 4725<br>Non-recommended indication found:<br>**N = 2748 (58%)**	N = 3848<br>Non-recommended indication found:<br>**N = 1969 (51%)**

[a] Contraindicated oral AT combinations: P2Y12 inhibitors combinations, anticoagulant combinations > 15 or 30 days, dual therapy or triple therapy with ticagrelor or prasugrel, combinations of 3 antiplatelets

[b] Dual antiplatelet therapy: aspirin–clopidogrel, aspirin–ticagrelor, aspirin–prasugrel

[c] Dual therapy: aspirin–VKA, aspirin–DOA, clopidogrel–VKA, clopidogrel–DOA

[e] Triple therapy: aspirin–clopidogrel–VKA, aspirin–clopidogrel–DOA

Abbreviations: AT: antithrombotics; DOA: direct oral anticoagulant; VKA: Vitamin K Antagonis

Exposure to oral AT combination was defined as the delivery to at least 2 oral ATs for at least 15 successive days. As sensitivity analyses, we defined oral AT combination as the delivery to at least 2 different oral ATs for at least 30 or 45 successive days. For this analysis, we considered all oral AT combinations (N = 5945), which differs from **Supplemental Table S5**, when we considered only the first oral AT combination

Supplemental Table S8: Risk of major bleeding in individuals with inappropriate oral AT combinations (N = 2580) versus appropriate oral antithrombotic (AT) combinations (N = 1932) as a reference during their follow-up (estimated by the fitted Fine and Gray model with death as competiting event).

Risk of major bleeding[$] **Variables (number in the class)**	**sHR (95% CI)**	**P value**
**Inappropriate oral AT combinations**[a]	0.83 (0.41- 1.68)	0.62
**Male gender**	1.01 (0.48-2.14)	0.97
**Age at study entry, years**		
Age[b] 65-79	0.81 (0.31-2.10)	0.08
Age[b] ≥ 80	2.30 (0.93-5.69)	
**Chronic kidney disease**	1.41 (0.30-6.70)	0.66
**Chronic hepatic disease**	2.05 (0.27-15.31)	0.48
**Coronary heart disease**	1.44 (0.67-3.09)	0.35
**Peripheral vascular disease**	0.39 (0.06-2.55)	0.32
**Non-valvular atrial fibrillation**	0.95 (0.18-4.83)	0.95
**Valvular heart disease**	1.24 (0.27-5.78)	0.78
**Stroke or arterial embolism**	1.42 (0.32-6.12)	0.84
**Venous thromboembolism disease**	3.24 (0.89-11.80)	0.07
**Hypertension**	1.20 (0.52-2.80)	0.67
**Diabetes**	1.38 (0.53-3.60)	0.51
**Anemia**	0.47 (0.06-3.42)	0.45
**History of major bleeding**	1.96 (0.23-16.89)	0.54

[$] Event of interest: hospitalization for major bleeding, N = 26; Event in competition: death = N = 25

[a] Reference group is appropriate oral AT combination

[e] Reference: 45–64 years old

Abbreviations: sHR: subdistribution hazard ratio; 95% CI: 95% confidence interval
